# Correspondence

**Published:** 1868-11

**Authors:** H. W. Alden

**Affiliations:** 200 E. 10th, St., N. Y


### ARTICLE VII.

NEW YORK, Oct. 21, 1868.

MESSRS. EDITORS:—

A process of hardening Rubber for dental use, without sulphur, has been perfected by an eminent chemist in this city, and will be offered to the profession in a short time. It is intended to give every dentist who may become a stockholder in the Company being organized to manufacture the article, the right to use the same in their profession *without a license*, and to confine the sale of stock to the members of the profession, making it really a dental association, the profits from the business to be divided among themselves.

Yours &c.

H. W. ALDEN, 200 E. 10th, S*t*., N. Y

